# Potentiation Physiologically Proven

**Published:** 1893-09

**Authors:** Gustav Jaeger

**Affiliations:** Stuttgart, Germany


### POTENTIATION PHYSIOLOGICALLY PROVEN.

Prof. Dr. Gustav Jaeger, Stuttgart, Germany.

[Translated by B. Fincke, M. D. Continued from page 427.]

### V. Further Physiological Facts.

Fortunately there are still other phenomena by which the potentiation can be controlled than the formation of numbers by means of the chronoscope, and I have recognized mainly two as especially amenable to the senses.

(*a.*) *The Smell.*

I premise that there is no need of a special education, such as the art of a wine or tea-taster, but of something which presupposes nothing but the possession of a sense of smell in general. Also a knowledge in this department is not required; by which, however, I do not want to say that it would not be of the greatest advantage for the practical man in the department of Homœopathy—quite the contrary: since the chemical reaction in a mass of medicinal substances is on the whole impotent, and even with the most accessible does not reach beyond the 6th or 7th potency, here as in the wine and tea-trade, where chemistry also is impotent, the nose (and tongue) should be the supreme judge, but only then when by exercise for years one has become familiar with the odor and taste of the medicinal substances and their various potencies. But this—*i. e.*, the discrimination of the various medicines by means of the sense of smell—is here not concerned, only what the *sense of smell can* observe in relation to *the degree of potency of one and the same substance*, but of course only then when in these provings he guards against the so easily occurring *blunting of the sense of smell*, of which I have published the details at different times.

Since I made all my experiments with alcoholic potencies, I always had a distinct olfactory impression in which, of course, the alcohol was prominent. Now it is known that in an alcoholic beverage the fineness of the *bouquet* contained in it, the presence of *fusel oil* or other concentrated admixtures can be recognized very well by means of the sense of smell. I found, then, that this is just as possible with the alcoholic *medicine-potencies*, even with relatively odorless substances, such as the neutral salts investigated by me. The difference of smell of the *lower* and *higher* potencies is most distinct, as, perhaps, no experimenter after me will fail to perceive; the *first* smell uniformly *musty*, *heavy*, *fusely*, *unclean*, the latter *fresh*, *pure*, *fine*, *light*, and twice (with Ammon-mur. 12th potency, and Kali-phosph. 19th potency) my measure-account contains the remark, "smells good," because it was actually a peculiar fragrancy. Of these two cases I will not at all assert that they are free of doubt (accidental contamination), but, on the whole, I am sure in this matter, in all the seventeen salts, after overstepping the neuro-analytical indifference, the mentioned change took place in the olfactory impression—at the same time one of the hundreds of proofs that Neural Analysis is not a swindle and imagination. As said before, everybody will observe the change who has the habit on buying or before using spirituous liquors to prove them by means of the smell for their purity. At any rate, the change cannot escape any expert in this department, and he will accomplish even more, but only under certain conditions. I have made my potencies in *equal vials*, and noticed at the time that not only the disagreeable olfactory impression of the *lower potencies* decreased from potency to potency—of course not in equal degree and equally distinct in all the salts—but that also in the *higher* potencies a gradual change, a refining of the odor could be perceived. Among the readers there are certainly such with fine noses, and since I myself am deficient in this respect, it would be interesting to me if one or the other would undertake the after-proving of this matter and communicate with me. For the present I maintain: by means of some preliminary exercise one shall learn to distinguish *in praxi* with the nose, whether a *lower* or a *higher* potency of a medicine is at hand, for the difference is exactly the same as that of a *good* and *bad* brandy, and if a physician, or apothecary, or inspector can do this at least, they are much more certain. I have made no experiments with the *sense of taste*, for being a smoker, it has lost its fineness. Besides, for this purpose, another *medium* than alcohol should be taken, because it acts too strong upon the tongue. I believe the best way would be to use *water*, for which we have already a precedent. Aside, of course, from the pollution of the *drinking* water we have for *good* and *bad*, a denotation of taste, which, indeed, is taken more from the touching part of the sense of taste than from the actual chemical sense: *soft* and *hard*. Lower potencies will taste *hard*, higher *soft*, and the former the softer the more they are removed from the point of indifference. Especially, then, when as well in a medicine as in the drinking water, mineral substances are concerned, the comparison with the gustatory impression of the drinking water will easily appear. In this department co-operation would be very useful.

(*b*.) *Involuntary Motions*.

The numerical material which I have communicated in Sections II, III, and IV of this essay is the result of an influencing of the *voluntary motory processes*. That also changes of the *involuntary motions* under the influence of substances of different potency-degree occur, is not the result of my latest provings, but already deposited in my former publications.

The reader, indeed, there finds only a part of them described, viz.: the one which I have pointed out by the kymographion and sphygmograph. High potencies influence the involuntary trembling of free members and pulsation; they, therefore, can also be proved in this direction upon their physiological power, which furnishes a valuable control for the neuro-analytical proving by chronoscope. See *Entdeckung der Seele*, 3d ed., vol. II, cap. 4 and 5. I have this time not proceeded in this direction.

Further on I have communicated in my *Neural Analysis of the homœopathic attenuations* that on performing neuro-analytical measurements, especially when the higher potencies are measured, and the more frequent in the higher potency, *zero-acts* occur—*i. e.*, finger-pulls, in which the jump of the clock-hand intermits. I have at that time in my publication said nothing of these phenomena, and for this reason. (*a.*) My main object at that time was directed to the formation of numbers, and little weight and attention was placed upon the other phenomena. (*b.*) The twitchings to be described hereafter were not absent, but in none of the four provers they were as severe as they are now with *me*. I exercise the Neural Analysis now during ten years, and my experience is that these involuntary twitchings which appear in consequence of the zero-acts have become more distinct and extensive from year to year. This, indeed, for some longer time receded into the background, because I did not work with medicinal high potencies, but only at hygienic examination of merchandise, in which often enough also higher potencies, but always the lower ones were concerned. Only now, after the above investigations, I obtain a certain idea. (*c.*) In the measurement for the Neural Analysis of Homœopathic attenuations every one of the four gentlemen measured for himself alone. Only after the publication I had an opportunity of observing *other* persons (an assistant and a few pupils) in their measurements, and then I noticed the twitchings as a phenomenon worthy of all attention, and I can comprise my experiences of that time as follows: *Zero-acts* will occur with most men if they want to obtain numbers by smelling high potencies, but more extensive co-motions (*Mitbewegungen*), even spasms occur, only on more sensible individuals, and a continual practice favors this occurrence. This fact is of fundamental importance toward two sides.

(*a.*) Of course, not the least unexpected is in the circumstance that different persons do not react equally strong to a physiological influence of a certain strength. But this is just the point to be attended to, that one should not judge incorrectly and confound the concepts *qualitative* and *quantitative*, *subjective* and *objective*. What are we going to prove by our experiments? Answer: The *objective* changes which in the physiological action of the medicine-substances are produced by the attenuation, therefore change of *quantity*. This proving cannot be obtained in any other way than by means of a physiological *subject*. With this necessarily a *qualitative*, or, in other words, *subjective*, element joins with the result of the proving, for different subjects, especially in man, are *qualitatively different*. Therefore, when I say I experience twitchings as soon as in this or that substance the 15th potency has been reached, and somebody says, "this is subjective," he is in so far right as this action does not at all occur with all subjects, but all the same I am right to consider the matter as an expression of an *objective* and *quantitative* fact; for (1) if from twenty different substances every time at a certain potency-degree I get twitchings whilst at the next lower degree they just as regularly do not occur; (2) if the extension and strength of the twitchings or cramps increases with me at every succeeding potentiation no rational being can contend that by the potentiation a change of quantitative nature, and that in the direction of greater force-development has been called out in the *object*—*i. e.*, the *medicine*—for to maintain that this came merely from a qualitative subjective change in me would be sheer folly.

(*b.*) In point of fact the adherents of Homœopathy have very different views on potentiation; however, two currents may be distinguished, the one up the other downward. This fact is by the above illustrated in a quite distinct manner. If we deduct those who don't think it necessary to procure for themselves a conviction by experiments on their own body, the above-mentioned subjective differences of the sensibility and practice must necessarily make themselves apparent in those who make the experiments upon themselves. The *more sensible* natures in which high potencies produce twitchings become unconditionally adherents of the high potency. In this not only their sensibility but also the not always but mostly connected more lively temperament assists them which is not deterred by the additional labor of high potentiation and the manifold opposition with which—at least with us—the practical use of the high potencies is accompanied. On the other side stands the more *torpid* part of the provers. These cannot so easily interest themselves for high potencies, because they are not affected by them, and deem the whole additional labor to be superfluous, to which also they are seduced by their more torpid temperament.

(*c*.) The *practice* (*Uebung*) is still to be considered. It is a known fact that limbs which are used a great deal are easily affected by involuntary twitchings and spasms. The most common example is the *writing-spasm* with which people become afflicted who must write a great deal. This throws a light upon practice; whoever must write much practices the muscles and nerves during the practice. The change which the practicing produces in them is a decrease of the *resistances* which the *conduction* of *irritation* meets, and with it a greater sensibility of irritation. In this relation see the chapter "Uebung" in my *Lehrbuch der Allgemeinen Zoologie II Theil Allgemeine Physiologie* (Leipsig, 1878). (The school-physiologists, by the way, contain on things practically so important as "practice," "habit," nothing at all !)

Now physiology has for the reflexes discovered the law of *irradiation*. This consists in that, in regard to these either on rising of the irritative strength or on heightening of the reflex-irritation the reflex-actions jump over to other motoric territories, and that in a distinct order and gradation.

The equal phenomenon is also valid for the *involuntary motions*, where higher sensibility exists as well as in the more stable one, such as in sensitive persons or on weakening of conductive resistances consequent upon frequent use, as well as in the transient sensibility produced by nervous excitement, the motory impulse jumps over to other muscular nerves according to the same law of irradiation, and with the voluntary motion involuntary ones associate themselves. But to the point!

The involuntary co-motions (*Mitbewegungen*) in the neuro-analytic measurements have a precursor in the so-called *zero-acts*, hence these must be described first. Furthermore, this is the more necessary because I am firmly convinced that even the more torpid natures can bring themselves to this stage of beginning excitement when experimenting upon themselves. As above described I allow in the Neural Analysis 10 acts (finger-*pulls*, not *pressures*) act upon the clock, the times of which add themselves thereby. As long as objects are measured which give *paralytic numbers* therefore poisonous concentrated heavy substances, invariably every finger-pull corresponds with a jump of the hand of the clock.

The same happens also when indifferent things or nothing is measured—*i. e.*, when rest-numbers are formed. This, however, is only then valid when one is actually in that repose which is presupposed for the practice of Neural Analysis, and the appearance of a zero-act on measuring rest-numbers, therefore, is for the practical neuro-analytician the surest sign that he is not at rest, but an *excitement* prevails which he unconditionally must remove or allow to fly off before he continues to measure.

On the other hand, the appearance of zero-acts in the more sensible natures is a regular occurrence as soon as they measure substances which render *animating numbers* and the explanation is that one or the other of the finger-pulls does not act on the clock, produces no jumps of the hand. (I here refer to my description in *Neural Analyse der homœop. Verdünnungen*, p. 6.) Generally this is the case with me if the decade-number indicates more than 30% animation, but it can also occur earlier.

If now we prove a series of potencies as I now again did with seventeen different substances and with one Kalicarbon twice with variation of the method, the invariable phenomenon occurs that under increase of the potency-degree the number of the zero-acts *increases*, which I have already mentioned in my *Neural Analyse d. hom. Verd.*

(*c.*) *Further Physiological Facts.*

What associates itself next in the further course is, with me, a slight drawing in the muscles of the measuring arm, and then I may be sure that in the next potency the further phenomena will appear which are distinguished *by two forms*, being at the same time two *stages*.

(1.) Mere *co-motions*, *twitchings:* whilst otherwise nothing responds to the shock of will than a pull of the finger, the experimenter can observe not only upon himself but also upon other persons further motions coinciding with the finger-pull, and here a distinct series of stages appear: *first stage:* a jerk of the whole measuring hand; *second stage:* beside hand-jerk, twitching of the whole measuring arm; *third stage:* in addition nodding of the head; *fourth stage:* now twitching of the flexor muscles of finger, hand, arm, head, trunk, and of the other arm; *fifth stage:* also the legs are contracted in jerks; *sixth stage:* the jerk of the legs is so sudden and short that on falling back they strike the floor with their full weight.

(2.) Actual *tonic spasm* first occurring in the measuring finger, mostly, however, immediately in the whole hand, and then also in the arm, then extending in the highest degree almost over all the muscles of the body, so that the experimenter is a startling sight to a second observer.

Here now clearly appears the connection between *zero-act* and *spasm* and the explanation of the first. During the continuance of the spasm in spite of all will-power, one is not able to exert such a finger-pull that the hand of the clock jumps, therefore the single *zero-acts* at the lower potencies are nothing but the first premonition of beginning spasmodic actions; the spasm is only limited to the measuring finger and then lasting such a short while that it suffices only for suppression or suffieient weakening of a single finger-pull. Later on when the spasmodic action extends also over further muscular regions, it also increases in duration so that several finger-pulls remain inactive. In the highest stage this can go so far that all the ten finger-pulls of the decade do not "come off," which in my numerical language I call the *great zero*, and in the case easily to be watched over when of the ten finger-pulls only one has acted upon the clock I call the *great one.* A more exact analysis of these phenomena would lead us too far and is not necessary for our purpose.

